# Cognitive Training Enhances Auditory Attention Efficiency in Older Adults

**DOI:** 10.3389/fnagi.2017.00322

**Published:** 2017-10-04

**Authors:** Jennifer L. O’Brien, Jennifer J. Lister, Bernadette A. Fausto, Gregory K. Clifton, Jerri D. Edwards

**Affiliations:** ^1^Department of Psychology, University of South Florida St. Petersburg, St. Petersburg, FL, United States; ^2^Department of Communication Sciences and Disorders, University of South Florida, Tampa, FL, United States; ^3^School of Aging Studies, University of South Florida, Tampa, FL, United States; ^4^Department of Psychiatry and Behavioral Neurosciences, College of Medicine, University of South Florida, Tampa, FL, United States

**Keywords:** aging, cognitive training, auditory cognition, attention, event-related potentials

## Abstract

Auditory cognitive training (ACT) improves attention in older adults; however, the underlying neurophysiological mechanisms are still unknown. The present study examined the effects of ACT on the P3b event-related potential reflecting attention allocation (amplitude) and speed of processing (latency) during stimulus categorization and the P1-N1-P2 complex reflecting perceptual processing (amplitude and latency). Participants completed an auditory oddball task before and after 10 weeks of ACT (*n* = 9) or a no contact control period (*n* = 15). Parietal P3b amplitudes to oddball stimuli decreased at post-test in the trained group as compared to those in the control group, and frontal P3b amplitudes show a similar trend, potentially reflecting more efficient attentional allocation after ACT. No advantages for the ACT group were evident for auditory perceptual processing or speed of processing in this small sample. Our results provide preliminary evidence that ACT may enhance the efficiency of attention allocation, which may account for the positive impact of ACT on the everyday functioning of older adults.

## Introduction

Hearing loss is a distressing impairment that becomes increasingly prevalent with age, affecting 30% of adults aged 65 to 74 and almost 50% of older adults over the age of 75 (National Institute on Deafness and Other Communication Disorders [NIDCD], 2010). Hearing loss causes speech perception difficulties (Humes et al., 2012) and has been linked to subsequent cognitive impairment (Lin, 2011; Lin et al., 2011a,b,c, 2013; Harrison Bush et al., 2015) as well as increased social isolation (Mick et al., 2014), reduced quality of life (Ciorba et al., 2012), increased risk for depression (Li et al., 2014), and reduced engagement in independent activities of daily living (Dalton et al., 2003). Because hearing loss results in effortful auditory processing in normal aging (Pichora-Fuller et al., 2016), remediation of auditory processing may improve cognition and enhance quality of life in older adults. The current study investigates the efficacy of auditory cognitive training (ACT) to improve underlying neurophysiological mechanisms of auditory perception and cognition.

Incipient, age-related hearing loss has been conventionally corrected with hearing aids. Despite improved audibility provided by current hearing aid technology, hearing aids alone often do not compensate for decreased ability to comprehend meaningful auditory stimuli among background noise. Speech perception, for instance, depends not only on intact auditory function but also intact cognitive function including the ability to attend to relevant stimuli, to process incoming stimuli, and to maintain new information all while actively manipulating temporarily stored stimuli. These cognitive functions all appear to decline in normal aging (for reviews, see Akeroyd, 2008; Arlinger et al., 2009). Thus, while hearing aids can enhance audibility, concomitant and subsequent age-related cognitive decline may persist, impairing older adults’ ability to process meaningful sound in challenging listening situations.

There is growing evidence that cognitive training programs can ameliorate or minimize age-related sensory and cognitive decline through neuroplastic change (Smith et al., 2009). However, the neural mechanisms underlying these changes are still relatively unknown (although see O’Brien et al., 2013, 2015). The purpose of the current study is to investigate the neural mechanisms underlying cognitive gains following auditory-based cognitive training.

The current study employs computerized ACT in an attempt to improve auditory perception and cognition (attention, speed of processing) in older adults. ACT is a process-based training targeting certain neural circuits via perceptual information processing. Process-based training is hypothesized to lead to transfer to other tasks that engage the same or overlapping neural circuit(s) regardless of whether the other tasks were specifically trained (Jonides, 2004). ACT has a positive effect on behavior reflecting auditory perception, memory, attention, and speed of processing (Mahncke et al., 2006; Smith et al., 2009; Anderson et al., 2013) and there is some evidence for far transfer (Strenziok et al., 2014). However, the underlying mechanisms of these gains remain relatively unexplored. Most of the extant literature has measured efficacy of cognitive training behaviorally through neuropsychological and psychometric tests. Behavioral measures reflect combined effort stemming from several stages of processing (i.e., sensory, cognitive, and motor) and performance is influenced by outside factors such as motivation and physical function, making it difficult to draw conclusions about the neurophysiological processes underlying behavioral changes.

An alternate approach used in the current study is measuring event-related potentials (ERPs), which are averaged signals from electroencephalogram (EEG) time-locked to a perceptual and/or cognitive event. ERPs are reflective of ongoing brain activity and are particularly sensitive to the timing of mental processes (on the order of ms), such that early perceptual activity can be distinguished from post-perceptual cognitive processes (Luck and Kappenman, 2012). Of particular interest in the current study is the P1-N1-P2 complex and the P3b component. The P1-N1-P2 complex is a fronto-centrally occurring series of ERP components measuring the physiological response of the auditory cortex to a stimulus, reflective of the neural detection of sound (for a review, see Key et al., 2005). The effect of age on these components is unclear. Data from numerous studies show both increases and decreases to amplitudes and latencies with age depending on methodological differences such as the attentional demands of the task (for a review, see Čeponienė et al., 2008).

The P3b component is a posterior-parietal component thought to reflect the attentional resources allocated for categorization of a target (Donchin, 1981; Pfefferbaum et al., 1984; Kok, 2001). It is sensitive to target probability, with unexpected or deviant stimuli eliciting a larger P3b than stimuli occurring with a high probability. Effects of aging on the P3b show a more frontally distributed, attenuated P3b amplitude and longer P3b latency (Anderer et al., 1996; Polich, 1997). This is consistent with recent theories of cognitive aging (Davis et al., 2008; Park and Reuter-Lorenz, 2009) indicating that prefrontal cortex (PFC) processing is recruited to counteract sensory, cognitive, and physical brain changes associated with normal aging. O’Brien et al. (2013) demonstrated an increase in older adults’ parietal P3b amplitude following process-based training in the visual modality compared to no-contact controls. Supporting evidence shows that P3b latency decreases after visual training are associated with better Useful Field of View performance (O’Brien et al., 2015).

In the present study, we predicted that ACT would result in improved attention consistent with previous evidence (Smith et al., 2009) and reflected cortically by a change in parietal P3b amplitude. Speed of processing improvements would be reflected in changes to parietal P3b latency. We also predicted that a frontal P3b would be present for all participants at baseline and would change following training. Also, if ACT impacts early cortical perception of auditory stimuli, we predicted a change to one or more components in the P1-N1-P2 complex in the form of amplitude or latency shifts, or a combination of both. Behavioral measures of cognition (Cognitive Self-Report Questionnaire, CSRQ) and speed of processing (Time-compressed speech, TCS) were included as corresponding evidence of neurophysiological changes. Changes in neurophysiological measures were predicted to correspond with changes in behavioral measures as a result of training.

## Materials and Methods

### Participants

Twenty-four experimentally naïve healthy older adult subjects (17 female, mean age = 70.88 years, mean years of education = 15.29) participated in exchange for cognitive training (see **Table 1** for demographic information). Participants were recruited from a list compiled of older adults who contacted the lab in response to a newspaper article or an ad placed in local media. This study was carried out in accordance with the recommendations of the University of South Florida institutional review board with written informed consent from all subjects in accordance with the Declaration of Helsinki. The protocol was approved by the University of South Florida institutional review board.

**Table 1:** Summary statistics for participants by group.

Measure	Trained (*n* = 9)		Control (*n* = 15)	
	*M %*	*SD*	*M %*	*SD*
Age (years)	69.69	7.66	71.60	8.29
Sex (female)	[7]		[10]	
Race (Caucasian)	[9]		[13]	
Education (years)	15.00	2.06	15.47	2.72
PTA of right ear	11.85	10.75	26.22	14.27
PTA of left ear	16.67	6.92	29.44	14.70

*PTA, pure tone average. Count in brackets.*

### Inclusion and Exclusion Criteria

Participants were required to: have a Mini-Mental State Examination (Folstein et al., 1975) score of 24 or greater (no severe cognitive impairment or dementia), have no self-reported neurological disorders, major strokes, or head injuries, have sufficient hearing (pure tone hearing thresholds <70 dB HL at 1000 and 2000 Hz in both ears), be a native English speaker, be available and willing to commit to the time and travel requirements of the study (maximum 22 visits), not be concurrently enrolled in another cognitive or training-related study, and not have previously completed a cognitive training program before participating.

### Group Assignment

Training-eligible participants were randomly assigned to computer-based ACT using Brain Fitness© (*n* = 9) or a no-contact control group (*n* = 15). During recruitment, participants were informed that they would be receiving cognitive training either immediately after baseline testing or subsequent to a second testing session 10 weeks after their baseline session. Chi-square analysis showed no significant differences between groups based on sex, *p =* 0.668. Independent samples *t*-tests revealed no significant differences between the groups in age or education, *t*s < 1.

### Measures

#### Audiometric Testing

A standard comprehensive audiometric evaluation was completed (American Speech-Language-Hearing Association, 2005) for both ears at octave frequencies between 250 and 8000 Hz to determine sufficient hearing to discern testing and training stimuli (pure tone hearing thresholds < 70 dB HL at 1000 and 2000 Hz in both ears). Testing was completed in a single-walled sound-treated booth suitable for audiometric testing. A three-frequency pure tone average (PTA) was calculated for each ear for using thresholds measured at 500, 1000, and 2000 Hz. PTAs lower than 25 dB are considered within normal hearing limits, 26–40 dB constitutes mild hearing loss, and moderate hearing loss at 41–55 dB. Participants in the trained group had PTAs ranging from 0 to 27 dB, significantly lower than those in the control group, *p*s < 0.025, which ranged from 5 to 55 dB (see **Table 1** for more descriptives).

#### Auditory Oddball Task

Frequent pure tone stimuli were presented (80% of the time) at 1000 Hz; oddball pure tone stimuli were presented (20% of the time) at 1500 Hz. Participants indicated the presence of an oddball stimulus by pressing a key on a computer keyboard. The task contained 8 blocks of 60 trials each (12 oddballs made up 20% and 48 frequents made up the remaining 80% of the stimuli presented) for a total of 480 stimuli (96 oddballs, 384 frequents) for each stimulus condition. The stimuli were 60 ms in duration and were presented at 80 dB SPL and the same wav file was used on each presentation. The task took approximately 15 min to complete.

#### Cognitive Self-Report Questionnaire (CSRQ)

The CSRQ is a 25-item self-report questionnaire comprising statements about an individual’s self-reported perceptions of hearing, cognition, and mood (Spina et al., 2006). Participants are asked to rate each statement (e.g., “I have had trouble hearing conversations on the telephone”; “I have felt I have a good memory”; “I have been in a bad mood” on a 5-point Likert scale from 1 “Almost Always” to “Hardly Ever.” The sum of all 25 items is calculated for a total score, with higher scores indicating more cognitive difficulties. The CSRQ has been reported to have excellent internal consistency (α = 0.91) and good 2-month test–retest reliability (*r* = 0.85) and has been used as a pre-and-post cognitive training tool to examine cognitive training effects on hearing, cognition, and mood in older adults (Spina et al., 2006).

#### Time-Compressed Speech (TCS)

The TCS is a low redundancy measure of auditory processing speed in which speech is digitally accelerated (compressed) to resemble fast speech (Beasley et al., 1980). For the current study, the TCS stimuli comprised the Northwestern University Number 6 words. Fifty words spoken by a female were presented binaurally at a 65% compression rate. Immediately after each word presentation, the participant was asked to repeat the word, even if he or she was unsure of the answer. TCS performance was defined as the percent of words correctly recognized with higher scores indicating better performance. Performance typically decreases with age (Sticht and Gray, 1969; Gordon and Fitzgibbons, 2001). The TCS is a routinely used clinical measure for auditory processing deficits and has been previously validated in older adults (Letowski and Poch, 1996).

### Procedure

Participants completed a screening visit to determine eligibility for the study and a baseline assessment of behavioral tasks (detailed above). EEG was recorded at baseline during performance of an auditory oddball task (detailed above). After baseline assessment, participants in the cognitive training group worked on computerized training exercises with the goal of completing a minimum of 16 training hours. Participants completed the auditory training program Brain Fitness (Posit Science). The program consists of six adaptive auditory exercises that are aimed at enhancing speed and accuracy of auditory processing. **Table 2** describes each exercise. The exercises are designed to simulate realistic listening contexts in a progressive fashion, moving from simple to complex auditory stimuli across exercises. Within each exercise, the stimuli become less discriminable and duration of stimulus presentation decreases as performance improves.

**Table 2:** Brain fitness exercises.

Exercise	Description
Frequency sweeps	Identify order of tone sweeps; shorter & faster sweeps
Tell us apart	Discriminate speech syllables; decreasing differences between syllables and increasing speed
Match it	Identify and remember speech syllables; increasing number of items and speed
Sound replay	Remember and identify order of words; increasing number of words and speed
Listen and do	Remember and follow instructions; increasing complexity of instructions and speed
Story teller	Comprehend story and answer questions; increasing story length and speed

Training sessions were 60 min in duration, 2 days per week, for up to 10 weeks, based on prior study protocols of cognitive training (e.g., Edwards et al., 2002, 2005; Ball et al., 2007). On average, participants completed training in 62 days (Min = 56, Max = 70, *SD* = 6.01), missing at most 1 week between sessions. During each training session, individuals were required to take at least one 5-min break, and were allowed to take additional breaks as necessary. Based on prior findings that the interval between sessions could vary without affecting efficacy (Vance et al., 2007), participants could skip training days if necessary, although frequent or extended missing of sessions was discouraged. Participants were supervised by a trainer in a group computer lab setting. The trainer was present to ensure on-task participation for the full session, as well as to clarify task instructions and handle any technical difficulties if necessary. On average, participants completed 18.78 h of training (Min = 14, Max = 20, *SD* = 1.99). Following training, participants repeated the same behavioral and auditory oddball tasks as completed at baseline.

Participants in the no-contact control group completed a second testing session 10 weeks following their baseline assessment, during which they repeated the same behavioral and auditory oddball tasks as completed at baseline. They were then invited to complete 10 weeks of training. We chose a no-contact control because previous research of cognitive training has revealed no differences between no-contact and social- and computer-activity control conditions (Wadley et al., 2006) on behavioral outcome measures.

### EEG Recording

Continuous EEG activity was recorded from 64 Ag/AgCl electrodes at standard 10/20 locations using Neuroscan^TM^ (SCAN version 4.3.1) with a SynAmps2 amplifier, with a vertex midline electrode position halfway between Cz and CPz as reference. For five trained and five control participants, continuous EEG activity was recorded using the NuAmps (NuAmp, Neuroscan, Inc., El Paso, TX, United States) single-ended, 40-channel amplifier according to the NuAmps International 10–20 electrode system using a Quikcap with sintered Ag/AgCl electrodes, and a continuous acquisition system (Scan 4.3 Acquisition Software, Neuroscan, Inc.). A right mastoid electrode was used as a reference. For all participants, four additional electrodes were placed on the outer canthus of each eye and on the supra and infraorbital ridges of the left eye to monitor eye movement and blink activity. Data was sampled at 1000 Hz with a 100 Hz low pass filter (time constant: DC). Electrode impedances were kept below 5 kΩ for most electrodes.

The experiment took place in a dimly lit, sound-attenuating booth. A Pentium 4 PC running E-Prime 1.1 (Schneider et al., 2002) recorded behavioral data and presented auditory stimuli. Responses were registered using a keyboard.

### Data Analysis and Predictions

Continuous EEG was high-pass filtered at a corner frequency of.1 Hz and low-pass filtered at a corner frequency of 30 Hz with a squared Butterworth zero-phase filter (12 dB/octave roll-off). Ocular artifact from eye movement and blinks were corrected for each subject by extracting the electroocular signals from the EEG. EEG for frequent and oddball trials was separated into epochs of 1000 ms (-200 ms before trial onset to 1200 ms after) and baseline corrected (-100 to 0 ms). Epochs in which EEG amplitude exceeded criteria of ±100 μV were rejected prior to averaging (*M* = 6%, *MAX* = 17%). Data were then averaged separately for each stimulus type (frequent, oddball). Data for the P3b component were re-referenced to averaged mastoids. Mean amplitudes and peak latencies were measured at parietal electrode site Pz and frontal electrode FCz for frequent and oddball stimuli in a 250 – 750 ms post-stimulus time window. Data for the P1-N1-P2 complex were re-referenced to a global reference. Mean amplitudes and peak latencies were measured at FCz for frequent stimuli in a 45 – 95 ms post-stimulus time window for P1, 105 – 155 ms post-stimulus time window for N1, and 225 – 275 ms post-stimulus time window for P2.

For each component, an Analysis of Variance (ANOVA) (or independent *t-*test where applicable) was first used to compare the two conditions at baseline, and repeated-measures ANOVA was used to examine training effects. All tests were two-sided and had an alpha level of.05. P3b analyses at Pz included within-participant factors of Testing Session and Stimulus Type, and the between-participants factor of Group. Effect sizes were calculated using omega squared (ω^2^). A significant Testing Session × Stimulus Type × Group interaction for P3b amplitude was expected to support the hypothesis that attentional allocation is enhanced post-training and the same interaction for P3b latency was expected to support the hypothesis that speed of processing is enhanced post-training. Frontal P3b analyses at FCz were conducted in the same manner with the same expected results. P1-N1-P2 analyses for frequent stimuli included the within-participant factors of Testing Session and the between-participants factor of Group. For all the above analyses, follow-up ANOVAs and *t*-tests were conducted to examine any significant effects within each subgroup.

Behavioral data from the oddball task were analyzed with repeated measures ANOVAs including within-participant factors of Testing Session and the between-participants factor of Group. The auditory oddball task was designed to be very easy to ensure high accuracy rates to preserve as many trials as possible for the electrophysiological analyses. Therefore, behavior from the oddball task was not predicted to change following training. Due to the small sample size, the sample was underpowered to be able to detect behavioral changes in the two auditory behavioral measures (CSRQ and TCS). However, prior research (Smith et al., 2009) has documented the positive effect of ACT on the CSRQ.

Finally, Pearson correlations were conducted to determine the relationship between any significant neurophysiological training gains and changes in auditory behavior measures. Alpha was set to 0.05. Mean amplitudes and peak latencies for all components are reported in **Table 3**. Behavioral scores for the CSRQ and TCS are reported in **Table 4**. Effects relevant to the proposed hypotheses are summarized below and all main effects and interactions are reported in **Tables 5–9**.

**Table 3:** Mean amplitudes and peak latencies for P3b and P1-N1-P2 at baseline and post-test per group.

	Trained (*n* = 9)				Control (*n* = 15)			
	Amplitude (μV)		Latency (ms)		Amplitude (μV)		Latency (ms)	
	Baseline	Post-test	Baseline	Post-test	Baseline	Post-test	Baseline	Post-test
P3b^a†^	5.14 (3.96)	3.12 (3.64)	473.78 (133.80)	397.89 (93.71)	3.12 (3.26)	3.21 (3.86)	509.07 (169.58)	445.07 (141.94)
P3b^a‡^	-0.17 (1.52)	-0.48 (1.03)	412.33 (204.69)	357.67 (143.44)	0.12 (1.04)	0.33 (0.81)	392.40 (177.92)	398.33 (168.62)
P3b^b†^	1.42 (4.93)	-0.84 (4.95)	531.33 (207.67)	505.89 (180.59)	2.75 (4.57)	2.41 (5.44)	414.07 (163.42)	475.27 (183.15)
P3b^b‡^	0.43 (1.08)	0.42 (1.10)	310.56 (162.90)	331.44 (154.46)	0.96 (1.09)	1.28 (1.54)	288.20 (72.31)	354.47 (187.56)
P1^b‡^	0.17 (0.53)	-0.16 (0.67)	61.67 (16.55)	66.89 (20.03)	0.14 (0.75)	0.23 (0.74)	68.40 (14.51)	66.93 (13.18)
N1^b‡^	-1.21 (1.11)	-1.67 (1.55)	122.89 (15.31)	124.67 (16.29)	-1.69 (1.55)	-1.85 (1.44)	128.07 (16.03)	124.47 (11.67)
P2^b‡^	2.50 (1.24)	2.21 (1.24)	243.22 (18.88)	249.89 (20.52)	1.73 (1.41)	1.89 (1.56)	245.20 (16.31)	247.73 (19.21)

*a, electrode site Pz; b, electrode site FCz. Standard deviations in parentheses; ^†^Oddball stimuli; ^‡^Frequent stimuli.*

**Table 4:** Mean behavioral scores for Cognitive Self-Report Questionnaire and time-compressed speech at baseline and post-test per group.

	Trained (*n* = 9)		Control (*n* = 15)	
	Baseline	Post-test	Baseline	Post-test
CSRQ^∗^	61.25 (15.39)	56.50 (15.20)	56.87 (10.64)	58.33 (10.70)
TCS	68.22 (8.63)	66.22 (11.81)	48.00 (12.67)	54.13 (20.11)

*CSRQ, Cognitive Self-Report Questionnaire; TCS, time-compressed speech at 65% compression; ^∗^Score for one training group participant is missing (*n* = 8). Standard deviations in parentheses.*

**Table 5:** Results of P3b amplitude at Pz repeated measures ANOVA.

	Overall			Trained			Control		
Variable	*F*	*p*	ω^2^	*F*	*p*	ω^2^	*F*	*p*	ω^2^
Group	0.07	0.797	-0.040	-	-	-	-	-	-
Testing session	8.66	0.008	0.014	30.12	0.001	0.070	0.42	0.529	-0.002
Stimulus type	23.70	<0.001	0.317	12.57	0.008	0.369	10.55	0.006	0.235
Group × testing session	14.58	0.001	0.025	-	-	-	-	-	-
Group × stimulus type	1.00	0.328	0.000	-	-	-	-	-	-
Testing session × stimulus type	5.37	0.030	0.011	11.03	0.011	0.036	0.06	0.817	-0.067
Group × testing session × stimulus type	4.01	0.058	0.007	-	-	-	-	-	-

**Table 6:** Results of P3b amplitude at FCz repeated measures ANOVA.

	Overall			Trained			Control		
Variable	*F*	*p*	ω^2^	*F*	*p*	ω^2^	*F*	*p*	ω^2^
Group	2.06	0.166	0.042	-	-	-	-	-	-
Testing session	3.50	0.075	0.009	11.82	0.009	0.044	0.00	0.988	-0.007
Stimulus type	0.40	0.532	-0.013	0.01	0.935	-0.057	1.29	0.274	0.010
Group × testing session	3.42	0.078	0.008	-	-	-	-	-	-
Group × stimulus type	0.59	0.452	-0.009	-	-	-	-	-	-
Testing session × stimulus type	5.46	0.029	0.016	7.38	0.026	0.040	0.66	0.432	-0.023
Group × testing session × stimulus type	1.60	0.220	0.002	-	-	-	-	-	-

**Table 7:** Results of P3b latency at Pz and FCz repeated measures ANOVA.

	Pz			FCz		
Variable	*F*	*p*	ω^2^	*F*	*p*	ω^2^
Group	0.26	0.615	-0.032	0.46	0.503	-0.023
Testing session	5.63	0.027	0.026	1.81	0.192	0.005
Stimulus type	4.38	0.048	0.040	25.41	<0.001	0.215
Group × testing session	0.83	0.372	-0.001	2.09	0.162	0.007
Group × stimulus type	0.24	0.631	-0.009	1.36	0.256	0.004
Testing session × stimulus type	0.92	0.347	-0.001	0.38	0.542	-0.003
Group × testing session × stimulus type	0.26	0.613	-0.006	0.25	0.624	-0.004

**Table 8:** Results of P1-N1-P2 Amplitudes at FCz Repeated Measures ANOVA.

	P1			N1			P2		
Variable	*F*	*p*	ω^2^	*F*	*p*	ω^2^	*F*	*p*	ω^2^
Group	0.63	0.435	-0.015	0.39	0.539	-0.026	1.01	0.327	0.000
Testing session	0.13	0.723	-0.017	0.86	0.364	-0.002	0.00	0.965	-0.006
Group × testing session	1.22	0.282	0.004	0.24	0.630	-0.008	1.03	0.321	0.000

**Table 9:** Results of P1-N1-P2 latencies at FCz repeated measures ANOVA.

	P1			N1			P2		
Variable	*F*	*p*	ω^2^	*F*	*p*	ω^2^	*F*	*p*	ω^2^
Group	0.33	0.574	-0.029	0.22	0.643	-0.034	0.00	0.990	-0.043
Testing session	0.14	0.712	-0.007	0.26	0.617	-0.009	1.23	0.280	0.002
Group × testing session	1.35	0.257	0.003	0.70	0.413	-0.004	0.30	0.592	-0.007

## Results

### Auditory Oddball Task Accuracy

Behavioral performance, accuracy for detecting the occurrence of oddballs, was above 99% (*SD*s below 2%) for both groups at both time points. ANOVA of behavioral accuracy revealed that performance did not significantly differ by group or by time, *F*s < 1. Reaction times to oddball stimuli also did not significantly vary by group or time point, *p*s > 0.200.

### Parietal P3b Amplitude

**FIGURE 1:**
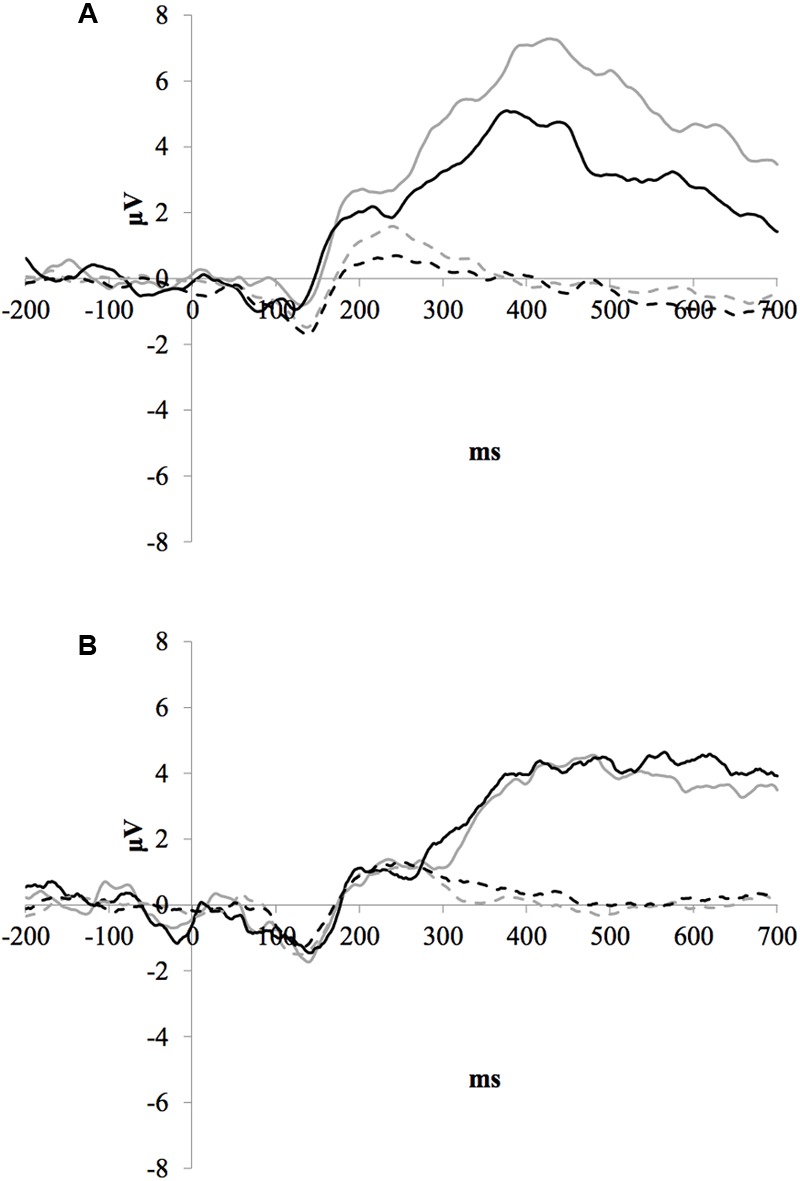
**(A)** Grand average ERP waveforms of the oddball (solid line) and frequent (dotted line) stimuli for the trained group at baseline (gray line) and post-test (black line) at electrode Pz. **(B)** Grand average ERP waveforms of the oddball (solid line) and frequent (dotted line) stimuli for the control group at baseline (gray line) and post-test (black line) at electrode Pz.

**Figure 1** illustrates the grand average ERP waveforms of the oddball and frequent stimuli for the trained and control groups at both testing points at electrode Pz. ANOVA of initial P3b amplitudes at baseline revealed that they did not significantly differ between groups, *F*(1,22) = 1.17, *p* = 0.292, ω^2^= 0.007. When comparing P3b amplitudes across time, the hypothesized interaction between Stimulus Type, Testing Session, and Group was marginally significant, *F*(1,22) = 4.01, *p* = 0.058, ω^2^= 0.007; there was also a significant interaction of Testing Session and Group *F*(1,22) = 14.58, *p* < 0.001,ω^2^= 0.025. Follow-up analysis of the trained group showed a significant interaction of Stimulus Type and Testing Session, *F*(1,8) = 11.03, *p* = 0.011, ω^2^= 0.036, reflecting a significant decrease in P3b amplitude to the oddball stimulus from baseline (*M* = 5.14, *SD* = 3.96) to post-test (*M* = 3.12, *SD* = 3.64), *t*(8) = 5.60, *p* = 0.001. There were no significant changes in amplitude across testing sessions after 10 weeks of no contact for the control group, *F* < 1.

Given the difference between the trained and control groups’ hearing PTAs at baseline, it is possible that the pattern of results were associated with this difference. To test this, we removed six participants from the control group who had average PTAs greater than 2.5 SDs from that of the trained group to conduct sensitivity analyses. Initial P3b amplitudes at baseline did not significantly differ between groups, *F*(1,16) = 1.32, *p* = 0.268, ω^2^= 0.017. When comparing P3b amplitudes across time, the hypothesized interaction between Stimulus Type, Testing Session, and Group was now not significant, *F*(1,16) = 2.68, *p* = 0.121, ω^2^= 0.005; however, there was still a significant interaction of Testing Session and Group *F*(1,22) = 13.20, *p* = 0.002,ω^2^= 0.030. Critically, there remained no significant changes in amplitude across testing sessions for the control group, *F* < 1.

### Frontal P3b Amplitude

**FIGURE 2:**
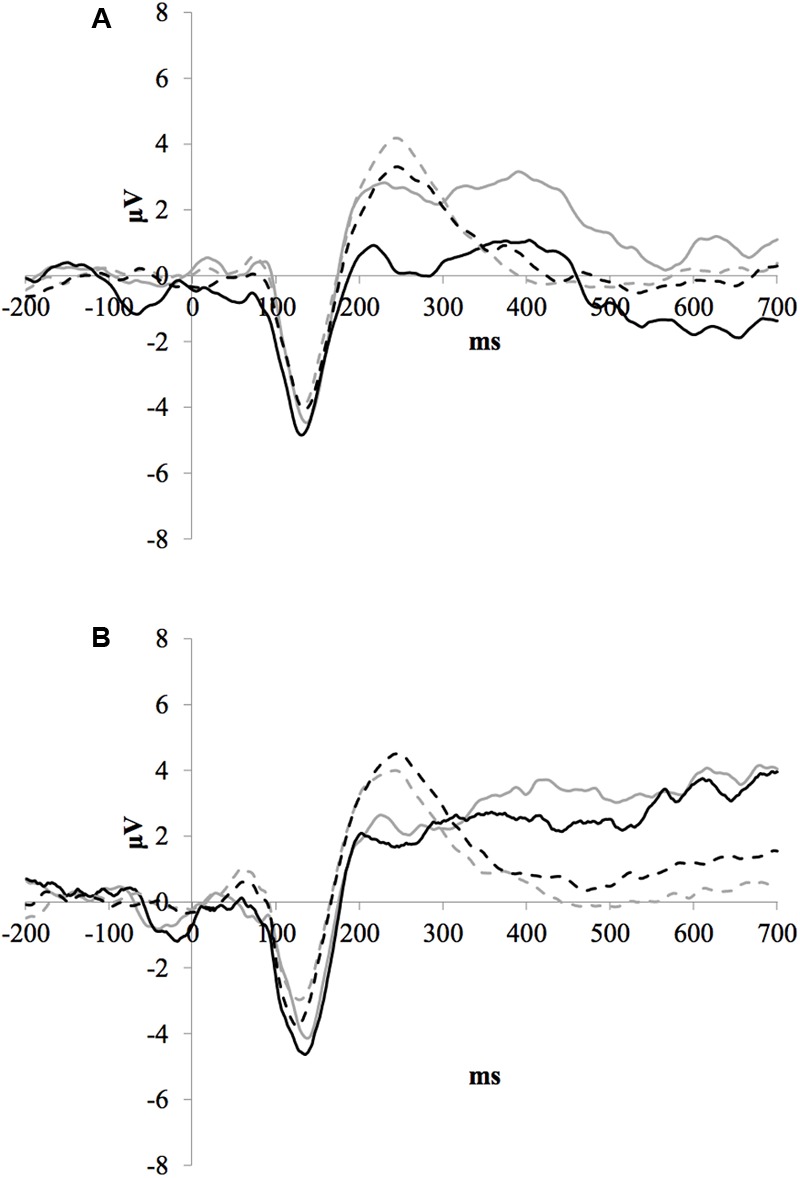
**(A)** Grand average ERP waveforms of the oddball (solid line) and frequent (dotted line) stimuli for the trained group at baseline (gray line) and post-test (black line) at electrode FCz. **(B)** Grand average ERP waveforms of the oddball (solid line) and frequent (dotted line) stimuli for the control group at baseline (gray line) and post-test (black line) at electrode Pz.

**Figure 2** illustrates the grand average ERP waveforms of the oddball and frequent stimuli for the trained and control groups at both testing points at electrode FCz. ANOVA of initial P3b amplitudes at baseline revealed that they did not significantly differ between groups, *F* < 1. When comparing P3b amplitudes across time, the hypothesized interaction between Stimulus Type, Testing Session, and Group was not significant, *F*(1,22) = 1.60, *p* = 0.220, ω^2^= 0.002. However, there was a significant interaction of Testing Session and Stimulus, *F*(1,22) = 5.46, *p* = 0.029, ω^2^= 0.016 and a marginally significant interaction of Testing Session and Group, *F*(1,22) = 3.42, *p* = 0.078, ω^2^= 0.008.

Given these effects and the *a priori* hypothesis of frontal shifts of P3b with aging, we conducted follow-up analyses to determine if significant P3b amplitude decreases occurred frontally as well for trained participants. Follow-up analysis of the trained group showed a significant interaction of Stimulus Type and Testing Session, *F*(1,8) = 7.38, *p* = 0.026, ω^2^= 0.040, indeed reflecting a decrease in P3b amplitude to the oddball stimulus from baseline (*M* = 1.42, *SD* = 4.93) to post-test (*M* = -0.84 *SD* = 4.95), *t*(8) = 3.28, *p* = 0.011. There were no significant changes in amplitude across testing sessions after 10 weeks of no contact for the control group, *F* < 1.

### P3b and Frontal P3b Latencies

ANOVA of initial P3b latencies at baseline revealed that they did not significantly differ between groups, *F* < 1. When comparing P3b latencies across time, there was a significant main effect of Testing Session, *F*(1,22) = 5.63, *p* = 0.027, ω^2^= 0.026. P3b latencies decreased from baseline (*M* = 447.85, *SD* = 132.63) to post-testing (*M* = 405.23, *SD* = 121.31), with no significant interaction of Group or Stimulus Type, *F*s < 1. There were no noteworthy significant latency effects at the frontal P3b.

### P1-N1-P2 Amplitude and Latencies

**FIGURE 3:**
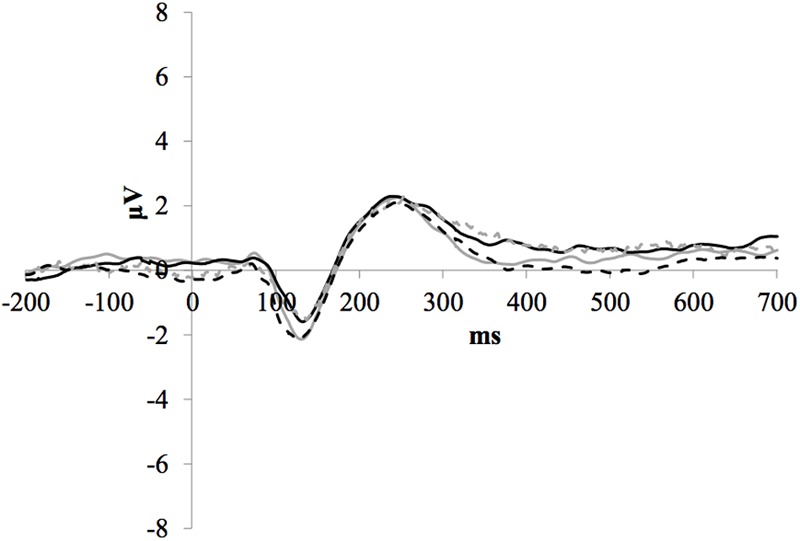
Grand average ERP waveforms of the frequent stimuli for the trained (solid line) and control (dotted line) groups at baseline (gray) and post-test (black) at electrode FCz.

**Figure 3** illustrates the grand average ERP waveforms of the frequent stimuli for the trained and control groups at both testing points. Comparison of initial P1-N1-P2 amplitudes and latencies at baseline revealed that neither significantly differed between groups, *ps* > 0.187. When comparing P1-N1-P2 amplitudes across time, there were no significant effects of time, group, or interactions for any of the three components, *ps* > 0.281. This was also the case for P1-N1-P2 latencies, *ps* > 0.256.

### ERP Correlations with Behavioral Data

To determine whether significant training effects on the P3b component corresponded with positive auditory behavioral gains, we first conducted a Pearson correlation between posterior P3b amplitude differences and differences self-reported perceptions of cognition (CSRQ) from baseline to post-test. A significant positive correlation was found between scores on the CSRQ and P3b amplitudes, with larger P3b amplitudes for the individuals with more self-reported auditory cognitive difficulties, *r* = 0.53, *p* = 0.010. We also conducted a Person correlation between posterior P3b latency differences and differences in our behavioral measure of auditory processing speed (TCS) from baseline to post-test, which was not significant, *p* = 0.501.

## Discussion

The goal of the current study was to elucidate the role of ACT on attentional and perceptual processing as measured by ERPs to help determine the underlying mechanisms of training gains and transfer. We provide electrophysiological evidence showing that engaging in process-based ACT can enhance attentional mechanisms in older adults.

### Training-Related Effect on P3b Amplitude

P3b amplitude is considered to reflect the attentional resources involved in comparing a significant or relevant event with an internal representation to categorize as a match or mismatch (Kok, 2001). Rare events, as a mismatch to internal representations, typically capture more attentional resources and result in larger P3b amplitudes than expected frequent events. Consistent with this, oddball stimuli (rare events) did elicit a larger P3b than frequent stimuli for both groups at both testing time points in the current study. However, oddball stimuli elicited a P3b following ACT that was smaller compared to pre-training amplitudes, while oddball amplitudes showed no change for controls across time. There was no significant difference between groups in baseline P3b amplitudes for oddball stimuli, but visual inspection shows a descriptively larger baseline amplitude for the trained group compared to the control group (see **Table 3, Figure 1**). Further study is needed to determine how baseline differences in cognitive abilities such as attention may impact neurophysiological measures after training.

While decreases in P3b amplitude are usually interpreted as diminished attentional resources, it is also possible that decreased P3b amplitude reflects diminished allocation due to the need for fewer attentional resources to categorize a stimulus. This interpretation suggests that ACT leads to an efficiency of attentional resource allocation and/or interaction with working memory updating. There is some prior evidence to support this hypothesis. Using meditation training, which promotes a broader attentional state, Slagter et al. (2007, 2009) showed decreased P3b amplitude to the first target in an attentional blink task following training compared to untrained controls. The attentional blink task requires attention to a rapid stream of stimuli and the subsequent report of two embedded target stimuli. Ability to report the second target in the stream is typically reduced if it appears within 500 ms after the first target (“attentional blink,” Raymond et al., 1992). A reduction in P3b amplitude to the first target suggests that training improved efficiency of attentional engagement such that resources were not solely devoted to the first target and were instead balanced between the two. In support of this, behavioral results showed a parallel reduction in the attentional blink following training. Mindfulness training has also been shown to reduce the P3b amplitude to incongruent words in a Stroop task possibly reflecting more efficient allocation of attentional resources during stimulus processing (Moore et al., 2012).

In a sample of young adults, Ben-David et al. (2010) showed a reduction in the late positive complex (LPC), a parietal response of which P3b is considered to be a subcomponent (Rushby et al., 2005), following an hour of auditory perceptual training of speech sounds. This was interpreted to reflect improvement in stimulus categorization and perceptual processes and possibly improvement in memory updating. Interestingly, Ben-David et al. (2010) showed the opposite effect of speech sound practice on the LPC, an increased LPC amplitude following training. They cite differences in experimental design between the two studies as a possible cause of amplitude reversal. Similar to this, O’Brien et al. (2015) reported larger P3b amplitudes following visual cognitive training compared to controls. In addition to being in a different modality, the oddball task used in their study was significantly more complex involving locating a perceptually different (tilted) target among an array of identical (horizontal) distractors. The possibility that visual and auditory process-based training impacts P3b amplitude differently needs to be further explored.

P3b amplitude was also significantly correlated with participants’ self-reported perceptions of their own auditory cognition (CSRQ). Participants who reported more cognitive difficulties had larger P3b amplitudes compared to those with fewer cognitive difficulties. ACT has previously been shown to result in CSRQ self-reported improvements by participants (Smith et al., 2009), suggesting that the neurophysiological changes occurring due to training may be behaviorally significant. Taken together, these findings show that a reduction in P3b amplitude can reflect efficiency in attentional resource allocation, such as flexibility in engaging and disengaging from relevant target stimuli. Attentional resource allocation following ACT is likely more efficient, resulting in fewer resources needed to categorize an oddball stimulus.

### Training-Related Effect on Frontal P3b Amplitude

Auditory cognitive training also resulted in a marginally significant decrease in frontal P3b amplitude compared to untrained controls. P3b activity shifts to a more frontal distribution with age and this shift has been interpreted as reflecting frontal lobe activity (for meta-analysis studies supporting this, see Friedman et al., 1997). Friedman and colleagues (Friedman et al., 1993; Friedman and Simpson, 1994; Fabiani and Friedman, 1995) report that older adults have two scalp distributions in response to auditory oddball stimuli – one frontal and the other parietal – suggesting that older adults activate frontal lobe processes to help encode these stimuli. Frontal areas are often activated in target detection tasks, corresponding with P3b generation (for reviews, see Polich, 2003; Fjell et al., 2007). As previously described, current theories of cognitive aging propose that PFC processing is recruited for cognitive tasks to compensate for parietal network functions that decline with age. Reduction in amplitude of the frontal P3b following training suggests that ACT potentially reduces the demand for PFC recruitment during attention processing.

### P3b Latency Changes

P3b latency did not show any change based on training as hypothesized but instead showed an effect of testing. Participants showed faster processing for both stimuli types during their post-test regardless of whether they were in the training or the control group. This is consistent with evidence that P3b latency reliability in an auditory oddball task is low in older adults both within a session (*r* = 0.07–0.24) and from 1 year to the next (*r* = 0.14–0.40) (Walhovd and Fjell, 2002). In addition, the correlation between P3b latency and behavioral auditory processing speed (TCS) was not significant.

Auditory cognitive training is designed to target speed of processing and has previously been shown to improve auditory processing speed in a sample of healthy older adults (Anderson et al., 2013) as well as in a sample of older adults with heart failure (Athilingam et al., 2015). It is possible that P3b latency as a measure of processing speed in the current study did not have enough power to detect an effect given the inconsistencies likely occurring within subjects. However, it is also possible that ACT primarily enhances allocation of attention rather than speed of processing.

### No P1-N1-P2 Changes

Auditory cognitive training did not impact auditory perceptual processing in the current study, as measured by P1-N1-P2 amplitude and latency. Enhancements in subcortical neural timing and speech perception following ACT have previously been observed using evoked potentials representing auditory brainstem responses to a speech syllable in noise (Anderson et al., 2013). It is possible that changes to early auditory perception following ACT are only measurable subcortically and that our later ERP measure was insensitive to these changes. The stimuli used in the current study – pure tones in quiet – were likely much easier to perceive than the speech in noise condition used in Anderson et al. (2013) study and it is therefore plausible that the insensitivity of our measure is due to a ceiling effect.

### Limitations

A significant limitation to this study is the small sample size. Randomized controlled studies involving cognitive training with older adults across a significant period of time are resource-intensive and prone to high levels of attrition for multiple reasons (e.g., voluntary withdrawal of consent, no longer meeting inclusion/exclusion criteria during course of study, poor adherence to training regimen). A large-scale, multi-site trial investigating the cognitive impact of ACT in older adults has been conducted (Smith et al., 2009), but this did not include any ERP measures to determine underlying mechanisms of training gains. To further investigate these underlying mechanisms, a study using neurophysiological measures similar in scope to Smith et al. (2009), utilizing intent-to-treat analysis modeling for attrition needs to be conducted.

Study demographics (primarily female, Caucasian, and well educated) also limit the interpretation of the current findings. Given the proposal that ACT targets speed of processing, and the current finding that ACT benefits attention and not speed of processing, multiple converging measures of these two functions should be included in future studies to clarify their role in training gains. Finally, the impact of ACT on functional outcomes as well as the long-term maintenance of training gains need to be investigated.

## Conclusion

The present finding of decreased P3b amplitudes following ACT reinforces the hypothesis that there is plasticity in the attentional control systems of older adults. Control over attentional resource allocation is vulnerable to age-related decline, but is shown here to be ameliorated by ACT. In light of previous findings demonstrating that portions of this training program result in improved cognition and transfer of gains to functional tasks (e.g., Smith et al., 2009; Anderson et al., 2013; Strenziok et al., 2014), our study is the first to provide preliminary neurophysiological evidence that ACT may particularly be enhancing the efficiency of attention allocation, which may account for the positive impact of ACT on the everyday functioning of older adults.

## Author Contributions

JO, JL, GC, and JE contributed to the conception and design of the work; JO and GC contributed to the acquisition of the data, JO, JL, BF, GC, and JE contributed to the analysis and interpretation of the data, drafted the work, JO, JL, BF, and JE revised the work critically for important intellectual content, JO, JL, BF, GC, and JE gave final approval of the version to be published and agree to be accountable for all aspects of the work.

## Conflict of Interest Statement

From June to August 2008, JE worked as a limited consultant to Posit Science, who currently markets the auditory cognitive training program used in this study. JE currently serves on the data safety and monitoring board of NIH grants awarded to employees of Posit Science. JE worked as a consultant to Wilson, Sonsini, Goodrich, and Rosati across an approximate 3 month period between May and August of 2015. The other authors declare that the research was conducted in the absence of any other commercial or financial relationships that could be construed as a potential conflict of interest.
